# Anthelmintic Prescribing Patterns of a Sample of General Practitioners from Selected Areas in the Colombo District of Sri Lanka

**DOI:** 10.4103/0970-0218.40876

**Published:** 2008-04

**Authors:** GSA Gunawardena, C Siriwardana, SR Paranavitane, MM Ismail, SD Fernando

**Affiliations:** Department of Parasitology, Faculty of Medicine, University of Colombo, Colombo, Sri Lanka; 1 Ceymed Healthcare Services (Pvt.) Ltd. 132, S. De S. Jayasinghe Mawatha, Nugegoda, Sri Lanka

**Keywords:** Anthelmintics, general practitioners, primary healthcare, Sri Lanka

## Abstract

General Practitioners (GPs) provide first contact care of children and pregnant mothers in the community. This study ascertained the prescribing pattern of anthelmintics to children and pregnant women by a sample of GPs from the district of Colombo. Two hundred medical practitioners engaged in full-time General Practice (100 urban and 100 rural), were selected randomly. A pre-tested interviewer-administered questionnaire was used to collect data. A total of 183 GPs aged between 26 and 72 years (median 38) participated with 94 coming from urban areas. Seventy percent of the GPs were male. Almost 13% of GPs from urban areas had a Postgraduate degree in comparison to 4.5% from the rural areas (*P* < 0.05). Over 50% of GPs had 6-20 years of service and over 30% treated 16-30 patients daily. Seventy-three percent of GPs from rural areas accessed health-related reading material either daily or weekly in contrast to only 40% from urban areas (*P* < 0.001). All GPs prescribed anthelmintics to children. Pyrantel pamoate was the preferred anthelmintic used for children by both groups. Approximately 55% and 64% of GPs from urban and rural areas, respectively, prescribed anthelmintics during pregnancy. A majority of GPs prescribed drugs after the first trimester. However, 25% from urban areas gave drugs during any trimester (*P* < 0.001). Regression analysis revealed that GPs with postgraduate qualifications, those having frequent access to health-related material and those seeing more than 30 patients daily, prescribed anthelmintics to pregnant women more often. Although routine de-worming of pregnant women and children should occur through government antenatal and well-baby clinics, and through the schools de-worming programme, it may not happen due to various reasons. Thus, GPs play a vital role in achieving good coverage of anthelmintics among children and pregnant women. Making available clear national guidelines on prescribing anthelmintics in Sri Lanka would improve the prescribing patterns of anthelmintics among GPs.

The World Health Organization is currently focusing on 13 neglected tropical diseases which debilitate, deform, blind and kill.(1) Soil-transmitted helminth infection is one such disease identified for control and prevention. At a unit cost of US$ 0.02 per treatment, a dramatic reduction in the prevalence of soil-transmitted helminthiasis can be achieved globally. Preventive chemotherapy aims at using anthelmintic drugs either alone or in combination as a public health tool for preventing morbidity due to more than one form of helminthiasis. The greatest challenge has been to expand regular anthelmintic drug coverage as a public health intervention to reach all at risk of morbidity due to helminth-induced diseases. Reducing the morbidity of human helminthiasis depends on policy decisions taken by health administrators. Efforts to reduce the morbidity will depend upon the dedication of the health professionals as well as the support of partners at the national level, who have committed resources to helminth control.(1)

Globally, 60% of victims of soil-transmitted helminthiasis are children.(1) In Sri Lanka, 89.7% of children living in the plantation sector of Sri Lanka are infected with at least one type of soil-transmitted nematode, while 41% of the women are infected with hookworm.(2,3) Further studies have indicated a prevalence of 16% of infection with *Ascaris lumbricoides* in teenaged girls living in urban areas.(4) The current recommendations for prescribing anthelmintics to children and pregnant women in Sri Lanka are that all children should be administered anthelmintics periodically after the age of 1 year and all pregnant women should receive anthelmintics after the first trimester of pregnancy.(5,6)

General Practitioners (GPs) provide first contact care in Sri Lanka. They play a pivotal role in the care of children and pregnant mothers in the community. However, the prescribing pattern of anthelmintics of the Sri Lankan GP has not been documented. Thus, ascertaining the prescribing pattern of anthelmintics by the GPs would be useful when planning out future strategies to achieve good control of intestinal helminthiases and prevent their dire consequences.

## Materials and Methods

### Study design

A cross-sectional study to assess the prescribing patterns of anthelmintics to children and pregnant women by medical practitioners engaged in full-time General Practice. The study was carried out for a period of 8 months from April to October 2006.

### Study setting

The study was carried out in the Colombo district within the Western Province of Sri Lanka. The Colombo district is divided into 12 Divisional Secretariat Divisions (DSDs). DSDs are administrative units below the district level with a median population of about 50,000 and an average surface area of 208 km^2^. Each DSD is further divided into administrative units known as Grama Niladhari (GN) Divisions. Six DSDs within the Colombo district were urban, while the other six were rural. An urban area was defined as a location within the purview of a Municipal or Urban Council, while the rural area was that within the administration of a Pradeshiya Sabha.(7) Either one or two GN divisions from each DSD were then selected randomly to represent the urban and rural communities. GPs practising in the area were identified with the help of the Medical Officer of Health of the area. Between 8 and 10 GPs were selected randomly from each GN division to make a total of 200.

### Study population

The study population comprised of 200 medical practitioners engaged in full-time General Practice (100 from urban and 100 from rural areas) from the Grama Niladhari Divisions of the Colombo district. Seventeen GPs who could not be interviewed by the Investigators were excluded from the study.

### Data collection

Data was collected by using a pre-tested interviewer-administered questionnaire. Informed verbal consent was obtained from all participants. Medical graduates were used as field interviewers to increase acceptability and reduce respondent burden. These individuals were extensively trained to administer the questionnaire by the Investigators. All data collected were checked by the Principal Investigator or a Co-Investigator at the end of the day. Any omissions were obtained the following day. Ten percent of the data entered was checked by an independent person.

### Data analysis

Data was computerized using spss 15.0 for Windows statistical software package. The effect of selected variables was analysed in relation to prescribing anthelmintics using the Chi-squared test. Logistic regression analysis was further applied to determine the predictive effect of the various factors on the usage of anthelmintics.

Ethical clearance was obtained from the Ethical Review Committee of the Faculty of Medicine, University of Colombo. The questionnaire was devoid of the names of participants, thus maintaining anonymity and confidentiality of the participants.

## Results

### General characteristics

The characteristics of the population are given in Table 1. A total of 183 GPs aged between 26 and 72 years (median 38) participated in the study, of which 94 individuals were from urban areas of the Colombo district. The majority of GPs from both urban (40%) and rural (34%) areas were of the 31-40-year-old age group; 70% of the GPs were male. Almost 13% of GPs from the urban areas had a Postgraduate degree from the Post Graduate Institute of Medicine, Sri Lanka, in comparison to 4.5% from the rural areas (*P* < 0.05). Over 50% of GPs had between 6-20 years of service and over 30% indicated that they treated 16-30 patients per day. Seventy-three percent of GPs from rural areas accessed health-related reading material on a daily or weekly basis in comparison to 40% of GPs from urban areas (*P* < 0.001). Among the urban GPs, 42.1% (*n* = 8) of those with ≤5 years of practice, accessed reading material daily, while the majority of those with more experience (>5 years) did so only monthly (*P* = 0.001). Such a difference was not seen among the rural practitioners.

**Table 1:** Characteristics of the population

Factor	Urban (*n* = 94) Number (%)	Rural (*n* = 89) Number (%)	*P*-value
Age in years			
≤ 30	19 (20.2)	1 (23.6)	0.575
31-40	38 (40.4)	30 (33.7)	
41-50	19 (20.2)	24 (27.0)	
>50	18 (19.1)	14 (15.7)	
Sex			
Male	66 (70.2)	61 (68.5)	0.806
Female	28 (29.8)	28 (31.5)	
Academic qualifications			
MBBS only	82 (87.2)	85 (95.5)	0.048
MBBS + postgraduate	12 (12.8)	04 (4.5)	
Number of years in practice			
≤5	19 (20.2)	23 (25.8)	0.627
6-20	52 (55.3)	44 (49.4)	
>20	23 (24.5)	22 (24.7)	
Average number of patients seen per day			
≤15	17 (18.1)	12 (13.5)	0.085
16-30	44 (46.8)	29 (32.6)	
31-50	18 (19.1)	27 (30.3)	
>50	15 (16.0)	21 (23.6)	
Access to health-related material			
Daily or weekly	35 (39.8)	65 (73.0)	<0.001
Monthly or less frequently	59 (62.8)	24 (27.0)	

### Pattern of prescribing anthelmintics to children

All GPs prescribed anthelmintics to children under the age of 12 years. Nearly 80% of GPs from urban and rural areas said that they would like to prescribe anthelmintics at 6-monthly intervals [Table 2]. However, the proportion of GPs who actually prescribed anthelmintics regularly at either 3-, 6- or 12-monthly intervals was below 40% in both areas. The age of commencement of treatment for intestinal nematodes varied widely with only 50% of urban and rural GPs commencing treatment when the child was 1 year of age. Pyrantel pamoate was the preferred anthelmintic used for children by both groups of practitioners [Table 2]. Although both groups of practitioners used pyrantel pamoate, a significantly higher number of rural practitioners prescribed it than their urban counterparts (*P* = 0.005). The chief reasons for prescribing anthelmintics to children were at the request of the mother, passage of worms in stools and pruritus ani [Figure 1]. Among the rural GPs (but not the urban), the numbers of years in practice contributed to different prescribing patterns among children. When mothers request for anthelmintics and when the children have pruritus ani, the majority of GPs with ≤20-year practice prescribed anthelmintics (almost 87.0%), while only 59.1% of those with >20-year experience did so (*P* < 0.05).

**Table 2:** Pattern of prescribing anthelmintics to children by General Practitioners

Factor	Urban Number (%)	Rural Number (%)	*P*-value
Prescribe anthelmintics (*n* = 183)			
Yes	94 (100)	89 (100)	
No	0	0	
Frequency of prescribing (*n* = 55)			
3 monthly	5 (14.7)	1 (4.8)	0.335
6 monthly	27 (79.4)	17 (81.0)	
Yearly	2 (5.9)	3 (14.3)	
Age at which anthelmintics are commenced (in months; *n* = 182)			
<12	13 (14.0)	15 (16.9)	0.501
12-18	55 (59.1)	58 (65.2)	
19-24	19 (20.4)	11 (12.4)	
>24 when required	6 (6.5)	5 (5.6)	
Regular prescriptions to child (*n* = 180)			
Yes	34 (37.0)	21 (23.9)	0.057
No	58 (63.0)	67 (76.1)	
Anthelmintics preferred for children (*n* = 183)			
Pyrantel pamoate (Pyrantin®)	50 (53.2)	65 (73.0)	0.028
Mebendazole (SPC/SPMC)	36 (38.3)	22 (24.7)	
Albendazole (SPC)	6 (6.4)	1 (1.1)	
Mebendazole (Vermox®)	2 (2.1)	1 (1.1)	

### Prescribing anthelmintics during pregnancy

Approximately 55 and 64% of GPs from the urban and rural areas, respectively, prescribed anthelmintics during pregnancy [Table 3]. Anthelmintics causing congenital abnormalities in the foetus and maternal ill-effects were the main reasons for not prescribing anthelmintics during this period. Some GPs also indicated that they do not give anthelmintics since the antenatal clinics run by the Government were supplying these drugs to the mothers. A majority of practitioners from both rural (86%) and urban (61%) areas prescribed drugs after the first trimester. However, a significant number of practitioners from urban areas (25%) gave drugs during any trimester (*P* < 0.001; Table 3). Differences in prescribing patterns during pregnancy were observed among the rural GPs according to their years of practice; 61.4% (*n* = 27) and 86.4% (*n* = 19) of those with 6-20 and >20-year experience, respectively, prescribed anthelmintics to pregnant women, while only 48.0% (*n* = 11) did so among those with less experience (*P* = 0.023). When worms are passed in stools, however, 56.5% (*n* = 13) with ≤5-year service prescribed anthelmintics, while only 36.4% (*n* = 16) and 18.2% (*n* = 4) did so among those with 6-20 and >20-year experience, respectively (*P* = 0.029). Such differences were not seen among the urban GPs.

**Table 3:** Pattern of prescribing anthelmintics during pregnancy by General Practitioners

Factor	Urban	Rural	*P*-value
	Number (%)	Number (%)	
Prescribe anthelmintics (*n* = 183)			
Yes	51 (54.3)	57 (64.0)	0.178
No	43 (45.7)	32 (36.0)	
Trimester at which first dose is given (*n* = 108)			
After first trimester	31 (60.8)	49 (86.0)	0.001
After second trimester	7 (13.7)	7 (12.3)	
Any trimester	13 (25.5)	1 (1.8)	
Drugs preferred in pregnancy (*n* = 108)			
Mebendazole (SPC/SPMC)	45 (88.2)	22 (38.6)	<0.001
Albendazole (SPC)	4 (7.8)	33 (57.9)	
Mebendazole (Vermox®)	2 (3.9)	2 (3.5)	

The drugs used in pregnancy differed significantly among the urban and rural practitioners. A significantly higher proportion of GPs from urban areas prescribed mebendazole, while the GPs from rural areas preferred to give albendazole (*P* < 0.001).

The passage of a worm in the stools was a key indication for giving an anthelmintic to pregnant women. Other reasons for prescribing the drug were the presence of pallor at the time of presentation or history of a poor socio-economic status.

Factors determining anthelmintic prescribing in pregnancy among the urban and rural GPs [Table 4] showed that academic qualifications, the average number of patients seen per day and access to health-related material appeared to play a significant role (*P* < 0.05).

**Table 4:** Factors determining anthelmintic prescribing in pregnancy among urban and rural General Practitioners

factor	Urban (*n* = 51)	Rural (*n* = 57)	χ^2^	*P*-value
Age in years				
≤30	10 (19.6)	10 (17.5)	0.74	0.863
31-40	18 (35.3)	20 (35.1)		
41-50	11 (21.6)	16 (28.1)		
>50	12 (23.5)	11 (19.3)		
Sex				
Male	35 (68.6)	34 (59.6)	0.94	0.332
Female	16 (31.4)	23 (40.4)		
Academic qualifications				
MBBS only	41 (80.4)	54 (94.7)	5.23	0.022
MBBS + postgraduate	10 (19.6)	03 (5.3)		
Number of years in practice				
≤5	11 (21.6)	11 (19.3)	0.22	0.898
6-20	25 (49.0)	27 (47.4)		
>20	15 (29.4)	19 (33.3)		
Average number of patients seen per day				
≤30	30 (58.8)	18 (31.6)	8.09	0.004
>30	21 (41.2)	39 (68.4)		
Access to health-related material				
Daily or weekly	24 (47.1)	49 (86.0)	18.60	<0.001
Monthly or less frequently	27 (52.9)	08 (14.0)		

Multivariate analysis of factors pertaining to prescribing of anthelmintics (such as whether from urban or rural area, number of patients seen per day, access to health-related material and academic qualifications), considering both urban and rural GPs together, revealed that a significant proportion of GPs with postgraduate qualifications prescribed anthelmintics to pregnant women, four times more than those with only MBBS qualification. The GPs who had frequent access (daily or weekly) to health-related reading material as well as those seeing more than 30 patients daily were three times more likely to prescribe anthelmintics to pregnant women than the GPs who accessed health material infrequently or saw less than 30 patients per day, respectively [Table 5].

**Table 5:** Summary of logistic regression analysis of factors determining anthelmintic prescribing during pregnancy (dependent variable: nonprescribing anthelmintics)

Factor	Regression coefficient	Odds ratio	95% Confidence interval	*P*-value
Constant	−1.769	0.170		0.016
Academic qualification				
MBBS + postgraduate		1.000		
MBBS only	1.479	4.389	1.139-16.920	0.032
Average number of patients seen per day				
>30		1.000		
≤30	1.166	3.209	1.644-6.266	0.001
Access to health-related material				
Monthly or less frequently		1.000		
Daily or weekly	−1.232	0.292	0.152-0.559	<0.001

## Discussion

Medical Practitioners engaged in full-time General Practice are key stakeholders in the healthcare system of Sri Lanka. They provide a substantial portion of relatively good quality and lowcost primary healthcare.(8) At present, the number of registered GPs in the country is approximately 550 (data obtained from the College of General Practitioners). The number of male GPs in the country is approximately 85%,(9) which is also reflected in this study where a male preponderance is seen. Only a small percentage (13% urban and 5% rural) of the study population had postgraduate qualifications despite a Diploma in Family Medicine and an MD in Family Medicine being conducted by the Post Graduate Institute of Medicine in Sri Lanka.

The prescribing patterns in General Practice in Sri Lanka and their relationship to the essential drugs list have been studied. An analysis of the morbidity pattern of individuals seeking GP treatment in 10 clinics from the three districts of the Western province including Colombo revealed that clinical manifestations of helminthiasis was the sixth commonest cause for the encounter with a primary-care physician.(10)

Periodic administration of anthelmintic tablets to school-age children is now part of the policy of many well-designed school health programmes in countries which report a high transmission of soil-transmitted helminthiasis. This strategy was adopted at the World Health Assembly held in May 2001 to improve the health and well-being of school children.(11) Children as young as 12 months have been included in the de-worming activities. The resolution of the World Health Assembly in 2001 was to provide regular treatment to at least 75% of school-age children by 2010.(11) In Sri Lanka, the current practice is to de-worm school children in standards 1, 4 and 7 during the school medical inspection programme, which however, functions poorly, mainly due to nonavailability of drugs. Pre-school children are de-wormed at government well-baby clinics.

Children younger than 12 years of age account for 32.1% of all general practice consultations in Sri Lanka.(9) In this study, both urban and rural practitioners prescribed anthelmintics to children. Approximately 50% commenced treatment at 1 year of age following the recommendations currently operational worldwide.(12,13) Only about 30% of practitioners (37% urban and 24% rural) de-wormed the children at regular (3-, 6- or 12-month) intervals, although it is recommended that annual or more frequent de-worming of children should take place depending on the prevalence and intensity of infection in the area.(14)

The anthelmintic drug preferred by both urban and rural practitioners for children was pyrantel pamoate syrup (Pyrantin®). Pyrantel pamoate being a paralysing vermifuge does not permit migration of worms following de-worming; a risk associated with mebendazole, which causes worm death by selectively and irreversibly blocking the uptake of glucose by the worm, could be a possible reason for its higher use, in addition to the liquid form of the drug being easier to administer to children than tablets. The use of mebendazole, supplied by the State Pharmaceutical Corporation (SPC) or manufactured by the State Pharmaceutical Manufacturing Corporation (SPMC), was more among the urban than the rural practitioners (*P* = 0.048). A significantly higher use of Mebendazole 100-mg tablets, costing Sri Lankan Rupees (SLRs.) 0.43 (SPC) or SLRs; 0.70 (SPMC) per tablet (US$ 0.004-0.007), was seen among the urban practitioners, while the rural practitioners preferred pyrantel pamoate (costing SLRs. 78.00 (US$ 0.78) for 10 ml of 50 mg/ml suspension and 1.75/125 mg tablet (US$ 0.02), respectively; *P* < 0.05).

Hookworm and other helminth infections have long been known to have adverse effects on maternal health and pregnancy outcomes. When widely-used, safety-tested anthelmintic drugs became available, a significant policy change was implemented. In areas endemic for hookworm infection, de-worming after the first trimester of pregnancy has been demonstrated to be safe, resulting in the improvement of maternal health, birth weight and infant survival.(11,15,16)

*Necator Americans* infection is endemic in many parts of Sri Lanka and the proportion of pregnant women with anaemia was estimated to be as high as 60%.(17) In 1994, it was formally recommended by the Sri Lankan Ministry of Health that mebendazole be given to all pregnant women after their first trimester through government antenatal clinics without routine stool examination.(5) Fifty-four percent of urban and 64% of rural GPs followed these recommendations of prescribing anthelmintics in pregnancy; the rest, not prescribing this drug was mainly because of its being supplied free of charge by the antenatal clinics. However, it has been reported that a significant proportion of pregnant women do not receive this drug due to nonattendance, long queues and the nonavailability of drugs at the government antenatal clinics.(16)

The study done in 1999 in Sri Lanka by de Silva and others showed that although mebendazole therapy during pregnancy is not associated with a significant risk of major congenital defects in the foetus, it should be given after completion of the first trimester; a practice which 25.5% of urban and 1.8% of rural practitioners failed to follow. The prescribing patterns of anthelmintics during pregnancy differed significantly between the urban and rural practitioners. The urban practitioners preferred the cheaper anthelmintic mebendazole (SPMC, 500 mg tablet costing SLRs. 2.80 or US$ 0.03), while the rural practitioners prescribed albendazole (SPC, costing SLRs. 10.00 or US$ 0.10). Preference for albendazole by rural GPs could be due to their greater awareness of its better efficacy against hookworms which are generally more prevalent under rural conditions.(18,19) Both drugs are recommended for use only after the first trimester.

Further analysis of the factors determining anthelmintic-prescribing patterns in pregnancy among GPs indicated that academic qualifications, the frequency of access to health-related material and the average number of patients seen daily were significantly associated with the prescribing pattern after controlling for other variables; 19.6% of GPs from urban areas who prescribed anthelmintics during pregnancy had postgraduate qualifications as compared to 5.3% of rural practitioners. These GPs had a four-times-greater tendency to prescribe anthelmintics as compared to those with only MBBS qualification. Treatment strategies for intestinal nematodes are incorporated in the curriculum of the Diploma in Family Medicine conducted by the Post Graduate Institute of Medicine (Prospectus of the Diploma in Family Medicine).

A significantly higher proportion (73%) of GPs from rural areas accessed health-related reading material on a daily or weekly basis in comparison to 40% of GPs from urban areas. The frequency of access to health-related material among those prescribing anthelmintics to pregnant women was also greater among rural GPs (86 and 48% among rural and urban GPs, respectively). The average number of patients seen daily was more among rural GPs (68.5% of rural GPs seeing more than 30 patients per day as compared to 41.2% of urban GPs; Table 4). The rural practitioners seem to be facing more challenges, making them to seek information and they appear to be more informed on patient management than their urban counterparts. Overall, those GPs seeing more than 30 patients daily were shown to prescribe anthelmintics more readily to pregnant women than those seeing a lesser number of patients. The greater experience these GPs gain by administering to larger numbers of patients may be helpful in improving their anthelmintic-prescribing pattern.

The World Health Organization's policies for routine de-worming of children and pregnant women are determined after the prevalence and intensity of soil-transmitted helminth infections in the specific endemic area are confirmed. Then the appropriate de-worming strategy is identified. In Sri Lanka, although routine de-worming of pregnant women and children should occur through government antenatal and well-baby clinics, and through the schools de-worming programme, it may not happen due to various reasons, the primary one being that anthelmintics are not readily available in the state sector. Thus, GPs have a vital role in achieving a good coverage of anthelmintic usage among children and pregnant women. The generally accepted practice is to de-worm two to three times a year depending on the prevalence and intensity of helminthiasis in the community. GPs could make an assessment of the level of infection in their own community and de-worm accordingly. Making available clear national guidelines on prescribing anthelmintics in Sri Lanka would improve the prescribing patterns of anthelmintics among GPs. This will not only improve the health status of the urban and rural populations, but will also delay the development of resistance against these drugs by the helminth parasites.
